# Addition of telephone coaching to a physiotherapist-delivered physical activity program in people with knee osteoarthritis: A randomised controlled trial protocol

**DOI:** 10.1186/1471-2474-13-246

**Published:** 2012-12-11

**Authors:** Kim L Bennell, Thorlene Egerton, Caroline Bills, Janette Gale, Gregory S Kolt, Stephen J Bunker, David J Hunter, Caroline A Brand, Andrew Forbes, Anthony Harris, Rana S Hinman

**Affiliations:** 1 Department of Physiotherapy, School of Health Sciences, The University of Melbourne, Centre for Health, Exercise and Sports Medicine, Melbourne, Vic, Australia; 2 Health Change Australia, Sydney, NSW, Australia; 3 The University of Western Sydney, School of Science and Health, Sydney, NSW, Australia; 4 Medibank Health Solutions, Melbourne, Vic, Australia; 5 Royal North Shore Hospital, Rheumatology Department and University of Sydney, Sydney, NSW, Australia; 6 Department of Epidemiology and Preventive Medicine, School of Public Health and Preventive Medicine, and Melbourne EpiCentre, Monash University, University of Melbourne and Melbourne Health, Melbourne, VIC, Australia; 7 Monash University, Centre for Health Economics, Melbourne, Vic, Australia

## Abstract

**Background:**

Knee osteoarthritis (OA) is one of the most common and costly chronic musculoskeletal conditions world-wide and is associated with substantial pain and disability. Many people with knee OA also experience co-morbidities that further add to the OA burden. Uptake of and adherence to physical activity recommendations is suboptimal in this patient population, leading to poorer OA outcomes and greater impact of associated co-morbidities. This pragmatic randomised controlled trial will investigate the clinical- and cost-effectiveness of adding telephone coaching to a physiotherapist-delivered physical activity intervention for people with knee OA.

**Methods/Design:**

168 people with clinically diagnosed knee OA will be recruited from the community in metropolitan and regional areas and randomly allocated to physiotherapy only, or physiotherapy plus nurse-delivered telephone coaching. Physiotherapy involves five treatment sessions over 6 months, incorporating a home exercise program of 4–6 exercises (targeting knee extensor and hip abductor strength) and advice to increase daily physical activity. Telephone coaching comprises 6–12 telephone calls over 6 months by health practitioners trained in applying the Health Change Australia (HCA) Model of Health Change to provide behaviour change support. The telephone coaching intervention aims to maximise adherence to the physiotherapy program, as well as facilitate increased levels of participation in general physical activity. The primary outcomes are pain measured by an 11-point numeric rating scale and self-reported physical function measured by the Western Ontario and McMaster Universities Osteoarthritis Index subscale after 6 months. Secondary outcomes include physical activity levels, quality-of-life, and potential moderators and mediators of outcomes including self-efficacy, pain coping and depression. Relative cost-effectiveness will be determined from health service usage and outcome data. Follow-up assessments will also occur at 12 and 18 months.

**Discussion:**

The findings will help determine whether the addition of telephone coaching sessions can improve sustainability of outcomes from a physiotherapist-delivered physical activity intervention in people with knee OA.

**Trial Registration:**

Australian New Zealand Clinical Trials Registry reference: ACTRN12612000308897

## Background

Knee osteoarthritis (OA) is a common and costly chronic musculoskeletal problem that leads to pain, loss of function, reduced quality-of-life [1] and increased mortality rates [2]. Many people with knee OA also experience co-morbidities such as obesity, depression and cardiovascular disease that further add to the OA burden. Interventions that foster appropriate lifestyle behavioural change, particularly around physical activity, are important for chronic diseases such as OA. Physical activity, encompassing both structured exercise and incidental physical activity, is recommended by OA and general health guidelines because of its positive impact on disease outcomes *and* health status. Both muscle strengthening and aerobic exercise are effective in reducing pain and improving function in the *short-term* in patients with knee OA. Benefits, however, are generally not sustained because adherence to such exercise and physical activity typically declines over time. Interventions that facilitate sustainability of physical activity behaviours in patients with knee OA may achieve longer-term clinical improvements and reduce the risk and impact of associated co-morbidities.

Levels of physical activity in people with knee OA are relatively low compared with non-arthritic older people [3], with most failing to achieve minimum levels of aerobic activity recommended for cardiovascular health [4-6]. This may have several consequences. First, reduced physical activity significantly increases the risk for developing other major health problems such as heart disease, diabetes, and cancer [7,8]. Indeed a recent study showed that almost all people with lower limb OA had at least one co-morbid disease and these co-morbidities were related to greater pain and functional problems [7]. Second, there is a consistent graded relationship between lower physical activity levels and reduced functional performance in people with knee OA such that people who are less physically active tend to demonstrate poorer physical function [9]. Third, lack of physical activity can exacerbate OA-related physical impairments such as muscle weakness. These physical impairments may negatively impact on the disease course [10] and may be associated with an increased risk of functional decline [11,12]. Thus, improving physical activity levels in people with OA is an important management goal, not only for knee OA symptoms specifically, but also for overall health in general.

Considerable evidence supports the benefits of structured physical activity in this patient population with all clinical guidelines recommending exercise as a core part of treatment for knee OA [13-16]. A recent Cochrane review found consistent *short-term benefits* of exercise over education or no treatment, with small-medium effect sizes for pain and function similar to those seen with knee OA drugs [17] but with fewer contraindications and adverse effects [14]. Home-based exercise with some degree of therapist contact is an effective mode of exercise delivery [17]. Compared with more closely supervised programs, home programs are more convenient for participants, are feasible in community settings and are cost-effective for large populations, increasing their suitability as a public health approach [18].

Muscle strengthening exercises are important given that muscle weakness is almost universal in people with knee OA [19] and is related to higher pain levels and reduced function [20]. Our randomised controlled trials (RCTs) of quadriceps [21] and hip muscle [22] strengthening exercise, together with a recent systematic review that included 18 RCTs [23], confirm that strength training can improve pain and function by clinically meaningful amounts in people with knee OA. A further benefit of strength training is the resulting increase in incidental levels of physical activity that may come with increased muscle strength [24]. Whole-body activity such as walking can also improve pain and function in people with knee OA [17,18,25] and is beneficial for co-morbidities. Thus both muscle strengthening and whole body physical activity should be promoted for those with knee OA.

Despite consistent findings of short-term improvements in pain and function with exercise, effectiveness tends to decline once the intervention ceases [26,27]. Ongoing adherence is thus one of the most important factors determining the longer-term effectiveness of physical activity for OA patients [28,29]. A complex array of factors can influence adherence in people with knee OA including psychological factors, intervention-related factors, and disease- and illness-related factors [29]. Adherence can be improved by a number of different strategies, including receiving attention from health professionals [17], keeping an exercise log, having an individualised program based on patient preference and goals, receiving booster or refresher sessions and receiving support from a telephone coach [30]. These strategies can be incorporated into physical activity interventions to maximise adherence and hence improve long-term clinical effectiveness.

Telephone coaching sessions, commonly called “health coaching” have been widely used, particularly by the health management industry in the USA and, more recently, increasingly in Australia, to improve adherence to treatment recommendations and to facilitate health behaviour change for chronic disease prevention and self-management [31]. Some indication of the widespread use of telephone coaching in the USA can be gained from the 2009 Healthcare Intelligence Network survey [32] which reported that 86% of surveyed organisations used telephone health coaching as the primary delivery mode of services. In Australia, the Get Healthy Information and Coaching Service® (http://www.gethealthynsw.com.au) and the Work Safe Victoria’s *WorkHealth Coach* telephonic health coaching program (http://www.workhealth.vic.gov.au), are examples of population-based telephone health coaching services provided by State Governments. Both programs aim to reduce chronic disease risk factors in the population and target increasing physical activity.

Despite this widespread use of telephone coaching in practice, there are a limited number of RCTs that investigate the efficacy of particular telephone coaching interventions, which may be partly due to commercial-in-confidence considerations. A relatively recent systematic review evaluated whether telephone coaching changes physical activity behaviour in adults. A total of 16 RCTs involving both healthy participants and those with a chronic condition (although none with OA) [33] were included, and the review concluded that there is a solid evidence-base supporting the efficacy of telephone coaching for improving physical activity levels (structured and incidental). The review noted that telephone-delivered interventions are best supplemented with other components, such as face-to-face sessions or print material. All six studies that included a longer-term follow-up (more than 6 months after ceasing intervention) showed sustained improvements in physical activity over the long term. Interventions lasting 6 to 12 months and including 12 or more telephone calls produced the most favourable outcomes. Of note is that telephone coaching interventions can be successfully delivered by a range of people including health educators, nurses and physical activity specialists.

To our knowledge, there have only been three previous RCTs utilising telephone coaching interventions in patients with knee OA. All aimed to improve overall self management by targeting a range of patient behaviours such as medication adherence, weight loss, increased physical activity, stress management and improved sleep quality, and all evaluated only immediate post-intervention effects [34-36]. These studies demonstrated modest improvements in pain, function and/or health status among those receiving the telephone intervention and lend support to the premise that such interventions may be useful in knee OA. None of the interventions, however, primarily focused on physical activity. In addition, the telephone intervention was independent of input from a health care professional. It follows that focussed interventions and integration of telephone coaching into clinical care might further improve outcomes [36].

It is difficult to compare between telephone coaching studies and to generalise results due to differences in the definition of “health coaching”, the length of sessions, the training methods used and the conceptual design of the programs [31]. A recent integrative review of health coaching interventions [37] outlined essential criteria for effective health coaching interventions. They were: that the program used goal setting, motivational interviewing, collaboration with health care providers and had a program duration of 6–12 months.

Although further empirical evidence for telephone coaching interventions is required, the theoretical base for behaviour change support interventions has long been supported in the health psychology and health behaviour change literature. There are a number of theoretical behaviour change models that are commonly used as the basis for interventions - stages of change, positive psychology models, social cognitive theory, theory of planned behaviour and the implementation and intention model [32]. In a review of the literature, Gale and Skouteris [31] outline the three main processes required to facilitate health behaviour change. The first is to assist the person to form a behavioural goal intention. This relates to whether a person has the requisite knowledge and wants to change (i.e. do they have sufficient motivation to form an intention to change). The second is to help to convert that intention into action and maintenance and the final process involves effective communication of information between the patient and the health professional. In essence, this boils down to a number of simple questions that the patient asks themselves – Do I know what to do?, Do I want to do it? and Am I able to do it? From the clinician’s and researcher’s perspective the question becomes –are there techniques that can be applied to influence the patient’s answers to these questions?

The Health Change Australia (HCA) Model of Health Change™ aims to help clinicians apply the theoretical concepts of behaviour change to daily practice (http://www.healthchangeaustralia.com). It is a clinical practice decision framework for integrating patient-centred communication and behaviour change principles and processes into clinical practice and programs. The model was designed to address the three crucial components of facilitating behaviour change outlined above: build motivation, identify and address barriers to build self-efficacy, and build and maintain the therapeutic relationship. It provides practitioners with an evidence-based health behaviour change clinical pathway to complement usual clinical pathways for prevention and treatment of health conditions. Telephone coaching interventions can use the HCA Model of Health Change as a practice framework to guide their conversations and to collect data to track intervention and behaviour change processes. The approach draws on principles and techniques used in motivational interviewing, solution-focused coaching and cognitive behavioural therapy and so includes the key features of effective programs outlined by Olsen [37].

The primary objective of this pragmatic RCT is to evaluate the addition of telephone coaching, based on current behaviour change theory and knowledge and designed to support physical activity behaviour change, to a physiotherapist-delivered physical activity intervention on pain and physical function in people with knee OA.

Primary hypotheses:

H1: The physiotherapy plus telephone coaching intervention will be more effective in improving pain and physical function at 6 months than physiotherapy only.

Secondary hypotheses:

H2: The physiotherapy plus telephone coaching intervention will be more effective in improving pain and physical function at 12 months and 18 months than physiotherapy only.

H3: Greater improvements in physical activity levels, health-related quality of life and psychological parameters, as well as greater home exercise adherence and better participant-perceived response to treatment, will be found in the physiotherapy plus telephone coaching intervention group compared with physiotherapy only at 6, 12 and 18 months.

H4: The physiotherapy plus telephone coaching intervention will be more cost-effective at 6, 12 and 18 months when total knee OA-related costs are compared and related to the effects of the intervention.

## Methods/Design

### Trial design

**Figure 1:**
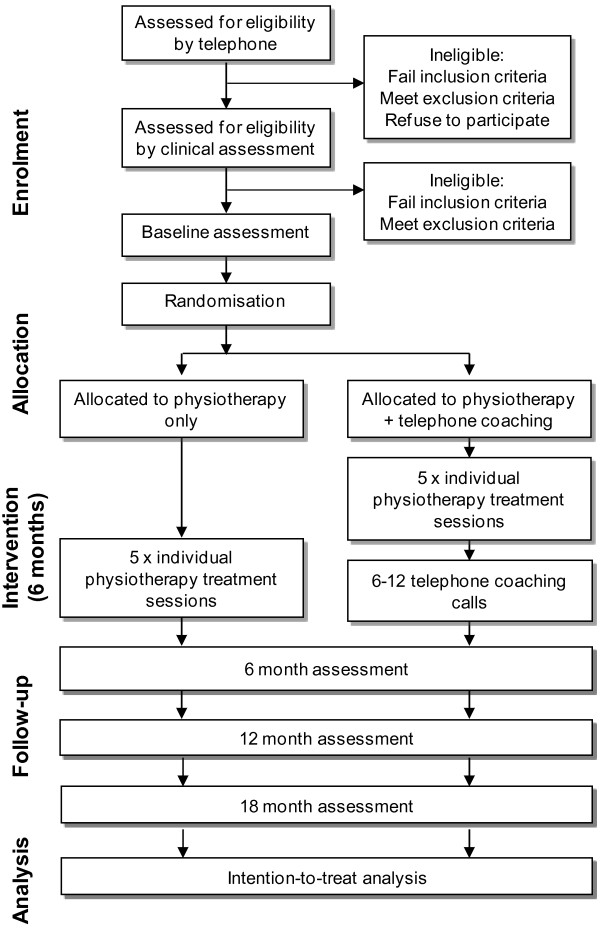
Flow diagram of study protocol.

Parallel-design 2-arm pragmatic RCT, with a 6-month intervention and outcomes assessed at 6, 12 and 18 months from baseline, with the primary outcome time point being 6 months. The study will be reported according to CONSORT guidelines for non-pharmacological studies [38] (Figure 1).

### Participants

One hundred and sixty-eight men and women aged ≥ 50 years with painful knee OA will be recruited from the community in metropolitan Melbourne and regional Victoria, Australia. Recruitment strategies will include (i) advertising through local clubs, community centers, newspapers, Arthritis Australia and University websites, radio, and Facebook; (ii) using our database of people with knee OA who were recruited from the community for prior studies not involving an exercise intervention and have given consent for future contact.

People will be eligible if they report average knee pain over the past week ≥ 4 on an 11-point numeric rating scale (0 = no pain, 10 = worst pain possible), and meet the American College of Rheumatology criteria for a clinical diagnosis of knee OA (any three of (i) 50 years or older, (ii) stiffness lasting less than 30 minutes, (iii) crepitus felt on passive or active movement of the knee, (iv) bony tenderness, (v) bony enlargement, (vi) no warmth to touch) [39]. Using a clinical diagnosis is consistent with primary care where x-rays should not be routinely ordered for diagnosis of knee OA. Only people who are classed as ‘sedentary’ or achieving ‘insufficient physical activity time’ according to the Active Australia Survey will be included [40,41].

Exclusion criteria will include:

i. inability to safely participate in moderate-intensity exercise as determined by the Sports Medicine Australia Stage I pre-exercise screening questions [42]

ii. currently undertaking regular lower limb strengthening exercises or receiving physiotherapy or other non-drug management for knee pain delivered by a health professional more than once within past six months;

iii. knee surgery or intra-articular corticosteroid injection within past six months;

iv. history of knee joint replacement on study knee or on waiting list for knee joint replacement;

v. systemic arthritic conditions or current or past (within four weeks) oral corticosteroid use;

vi. any other condition affecting lower limb function to a greater extent than their knee pain;

vii. unable to use/access a telephone;

viii. score ≥ 21 on the depression subscale of the Depression, Anxiety and Stress Scale.

People who have been on glucosamine, chondroitin and/or non-steroidal anti-inflammatory drugs will not be excluded. Participants will be asked to refrain from commencing exercise or other treatment for knee OA during the course of the study.

### Study procedure

Eligibility of prospective participants will be confirmed initially by telephone screening then by clinical examination by a physiotherapist. All eligible participants will be consecutively randomised into the physiotherapy only or the physiotherapy plus telephone coaching program. Assessments will be by self-report questionnaires at baseline (prior to randomisation), 6 months, 12 months and 18 months. In addition, 7-day objective recording of physical activity will occur at baseline, 6 months and 18 months, and health service usage and adherence data will be collected at 3-monthly intervals for 18 months (Table 1). All participants will visit a physiotherapist for 5 sessions over the 6-month intervention period. Telephone coaching sessions will be delivered 6–12 times during the 6-month intervention period. Participants will be advised to continue with their physical activity program during the unsupervised follow-up period and beyond. Ethical approval has been obtained from the University of Melbourne Human Research Ethics Committee (HREC No. 1137237). All participants will provide written informed consent prior to attendance for the clinical screening assessment.

**Table 1:** Summary of measures to be collected

**Primary outcome measures**	**Data collection instrument**	**Collection Points**
Average pain in past week	11-point numeric rating scale (NRS)	0, 6, 12, 18 months
Physical function in past 48 hours	WOMAC Osteoarthritis Index physical function subscale	0, 6, 12, 18 months
**Secondary outcome measures**		
Average pain on walking in past week	11-point numeric rating scale (NRS)	0, 6, 12, 18 months
Pain in past 48 hours	WOMAC Osteoarthritis Index pain subscale	0, 6, 12, 18 months
Perceived change overall	7-point ordinal scale	6, 12 and 18 months
Perceived change in pain	7-point ordinal scale	6, 12 and 18 months
Perceived change in function	7-point ordinal scale	6, 12 and 18 months
Physical activity levels	Active Australia Survey	0, 6, 12, 18 months
	Physical Activity scale for the elderly (PASE)	0, 6, 12, 18 months
	7-day ActivPAL^TM^ physical activity recording	0, 6 months
Health-related quality of life	AQoL2 questionnaire	0, 6, 12, 18 months
**Other measures**		
Self-reported psychological measures	Arthritis self-efficacy scale	0, 6, 12, 18 months
	Arthritis impact scale (AIMS2)	0, 6, 12, 18 months
	Mood, tension and thoughts subscales	
	Pain catastrophising scale (PCS)	0, 6, 12, 18 months
	Coping Strategies questionnaire (CSQ)	0, 6, 12, 18 months
	Depression, Anxiety and Stress scale (DASS)	0, 6, 12, 18 months
	Self-efficacy for physical activity scale	0, 6, 12, 18 months
	Barriers to physical activity scale	0, 6, 12, 18 months
	Benefits of physical activity scale	0, 6, 12, 18 months
	Brief fear of movement scale	0, 6, 12, 18 months
	Self regulation scale	0, 6, 12, 18 months
	Patient Health Questionnaire-9 (PHQ-9)	0, 6, 12, 18 months
Barriers and enablers to home exercises	Customised questionnaire	6, 12, 18 months
Adherence to intervention	Number of physiotherapy sessions attended	During intervention
	Number, timing and duration of telephone calls	During intervention
	Number of times home exercises performed	3, 6, 9, 12, 15, 18 months
	in past 2 weeks - questionnaire	
	Self-rated adherence to home exercise	3, 6, 9, 12, 15, 18 months
	Self-rated change in physical activity levels	3, 6, 9, 12, 15, 18 months
	Physiotherapist-rated participant adherence	6 months
	Telephone coach-rated participant adherence	6 months
Adverse events and harms	Log sheets	3, 6, 9, 12, 15, 18 months
Use of health services/co-interventions	Log sheets	3, 6, 9, 12, 15, 18 months
Willingness to pay	Questionnaire	6, 12, 18 months
Height	Collected by physiotherapist	0 months
Weight & waist circumference	Collected by physiotherapist	0, 6 months
Descriptive information	Questionnaire	0 months

### Blinding

Outcome assessment comprises self-reported questionnaires that will be completed by participants at home and returned to investigators by mail or email. The research assistants entering the data will be blinded. The physiotherapists, telephone coaches and participants are by necessity unblinded. The statistician will be blinded to group allocation until completion of the statistical analyses.

### Randomisation and allocation concealment

The randomisation schedule will be prepared by the study biostatistician (AF) using a computer generated random numbers table. Randomisation will be conducted by random permuted blocks of size 6 and 12, and stratified according to treating physiotherapist so that all physiotherapists deliver approximately equal numbers in each treatment group to control for physiotherapist variation. Participants allocated to physiotherapy plus telephone coaching will be randomly allocated one of the three telephone coaches. Participants will attend their preferred physiotherapist according to geographical convenience. Consecutively numbered, sealed, opaque envelopes containing treatment group and health coach allocation will be prepared by a researcher with no other involvement in the study. The envelopes will be stored in a locked location and will be opened in sequence to reveal group allocation by a researcher not involved in recruitment.

### Intervention providers

Thirteen physiotherapists who have at least 2 years post-graduate musculoskeletal experience and work in private clinics in metropolitan and regional Victoria will provide the physiotherapy intervention. Physiotherapists were chosen to deliver the physical activity intervention as they have expertise in exercise prescription and are key providers of structured physical activity in the community for people with knee OA. A clinical practice survey in the United Kingdom has shown that 100% of physiotherapists utilized exercise for this patient group [43].

Three registered nurses who complete training and mentoring from Health Change Australia to develop skills in providing behaviour change support will provide the telephone coaching for the study. None of the nurses will have training or experience in health behaviour change support or telephone coaching prior to involvement with this study.

### Interventions

#### Physiotherapy only

Participants will visit a physiotherapist for five 30-minute sessions over the 6-month intervention period: in weeks 1, 3, 7, 12 and 20. The physiotherapist will prescribe a home exercise program and advise the participant to increase their levels of general physical activity. Five physiotherapy visits was chosen as this number should be able to achieve improvements in pain and function [17] and importantly has translational potential into the Australian health setting given that the Federal Government Medicare scheme can currently fund up to five visits per annum to an allied health professional for patients with a chronic problem such as OA.

Over the five sessions, the physiotherapist will perform standardised assessment/re-assessments, develop a home exercise program and promote increased levels of general physical activity, including aerobic activity such as walking and incidental physical activity. The physiotherapist will also assist the participant to gain knowledge about OA and the benefits of physical activity. Participants will receive an information booklet which covers the following topics: condition and treatment information, exercise and physical activity, pain management, benefits of weight loss, relapse prevention and management, and facilitators for change (http://bit.ly/KaPPTW). They will also receive exercise handouts demonstrating the home exercises, a pedometer as an optional self-monitoring and motivational tool, and log sheets to record exercise and other physical activity if desired.

The home exercise program is designed to primarily strengthen the knee extensor and hip abductor muscles of the affected limb. Our research [22,44] and that of others [23] have shown lower limb strengthening exercises to be effective in improving pain and function in knee OA. The program will comprise a minimum of 4 and a maximum of 6 individualized lower limb exercises to be performed 3 times per week. All exercise programs will include at least 3 knee extensor strengthening exercises, and at least 1 hip abductor strengthening exercise selected from a pre-determined list (Table 2). The remaining optional 1 or 2 exercises can be chosen from other exercises on the list or be any exercise of the physiotherapist’s choice, in order to address an impairment or functional deficit related to the participant’s knee problem. Examples include functional drill or dynamic balance exercise(s), muscle stretch(es) or other lower limb muscle strengthening. The physiotherapist will select exercises and prescribe dosages for each exercise based on the assessment findings, including muscle strength, the participant’s pain and their perceived level of effort during performance of the exercise. Participants will be provided with elastic bands and/or ankle cuff weights for execution of the exercises if required. The physiotherapist will teach the participant the home exercises firstly by demonstrating the exercise and then supervising the participant performing the exercise, prescribe dosage, monitor progress and adjust the program as appropriate with the aim of progressing the exercises in intensity and/or difficulty over the 6-month intervention phase.

**Table 2:** Pre-specified list of exercises for the home exercise program

**Knee extensor strengthening:** Every program must include at least three of the following knee extensor strengthening exercises.			
**Knee extension**	Non weight-bearing	Seated knee extension (with resistance) with 5 second hold	Variation: Use appropriate level of resistance band – red through to black or ankle cuff weight
	Non weight-bearing	Inner range quads over roll (with resistance) in supine with 5 second hold	Variation: Use appropriate level of ankle cuff weight
**Straight Leg Raise**	Non weight-bearing	Straight Leg Raise in supine (with resistance)	Variations: Add 5 second hold
			Variation: Use appropriate level of ankle cuff weight
	Non weight-bearing	Straight Leg Raise in standing with resistance band at ankle	Variation: 5 second hold
**Resisted knee extension in standing**	Weight-bearing	Resisted inner range knee extension in standing (resistance band around back of knee)	Variation: Increase weight taken on arthritis leg until standing on one leg to do the exercise
**Partial Squats**	Weight-bearing	Partial wall squats with weight distributed bilaterally (feet approximately 30cm out from wall)	Variations: 5 second hold, more weight on arthritis leg
**Steps**	Weight-bearing	Step-ups (affected leg on the step, control knee straightening, lower to start position by controlling knee bending)	Variations: step height, holding extra weight (in hands or backpack)
	Weight-bearing	Forward touchdowns from a step (affected leg on the step, control knee bending to lightly tap floor in front with toes of non-affected leg, return to start by controlling knee straightening)	Variations: step height, holding extra weight (in hands or backpack), don’t touch down
**Sit-to-stand**	Weight-bearing	Sit to stand from a standard height chair without using hands/arms	Variations: chair height, hover above the seat without touching down, more weight on arthritis leg
**Forward-backwards exercise (with knee bend)**	Weight-bearing	Sliding (slide non-affected side foot along the floor to the front and then to the back, bend and straighten affected knee with control and neutral alignment)	
	Weight-bearing	Stepping (step non-affected side foot to the front and then to the back, bend and straighten affected knee with control and neutral alignment)	
**Hip abductor strengthening:** Every program must include at least one of the following hip abductor strengthening exercises.			
**Side-lying hip abduction**	Non weight-bearing	Side-lying bent-leg hip abduction (clams) with resistance band around knees	Variation: Use appropriate level of resistance band – red through to black or ankle weight
	Non weight-bearing	Side leg raise (hip abduction) with resistance	Variation: Use appropriate level of ankle cuff weight
			Do not use if painful hip OA.
**Standing hip abduction**	Non weight-bearing	Standing side leg side raises with resistance band	Variation: Use appropriate level of resistance band – red through to black or ankle weight
	Weight-bearing	Wall push standing on arthritis leg (non-affected leg bent at hip and knee, push thigh against a wall to activate hip abductor muscles)	Variation: Increase arthritis leg knee bend to 30°
**Side stepping**	Weight-bearing	Crab walk (side stepping) with resistance band around thighs or ankles	
**Hip abduction dips**	Weight-bearing	Hip abductor dips (standing on affected leg, lower non-affected leg by frontal plane pelvic tilting)	

A brief assessment will be performed by the physiotherapist at each physiotherapy session in order to ascertain any adverse effects (if any) that may have occurred with home exercises and to check quality and form of exercise performance. Progression of exercises is an essential component of the program and the findings from the assessment will help guide physiotherapists’ decisions regarding progression. Progression will be provided by varying the exercises including the type of exercise as well as the number of repetitions, load or degree of difficulty within an exercise. In order to gain strength, the level of effort experienced during each strengthening exercise will be self-rated as at least 5 out of 10 (hard) on a modified Borg Rating of Perceived Exertion (RPE) CR-10 scale designed specifically for strengthening exercise [45]. In addition, the resistance prescribed will aim to approximate a 10-repetition maximum level.

In order to minimise burden of exercise, only the study knee will be the focus of treatment and will be evaluated with respect to outcome measures. If participants have bilateral symptoms, the physiotherapist may choose exercises that are performed weight-bearing on both legs simultaneously to achieve bilateral strength gains within the constraints of the treatment protocol.

The physiotherapists will also discuss the disease-specific and general health benefits of increased levels of general physical activity, including both incidental physical and whole-body exercise. To facilitate increased incidental physical activity, an optional but strongly encouraged part of the physical activity intervention will be use of a pedometer. A pedometer (Omron HJ-005, Omron Healthcare Co, Kyoto, Japan) will be provided to each participant to allow them to monitor their incidental physical activity throughout the day, and receive immediate feedback on their progress towards general activity goals. Pedometers have been previously used successfully in OA populations for such self-monitoring and motivational purposes [18,25]. The physiotherapist will educate the participants in the use of the pedometer and discuss options for achieving increases in daily step count and setting relevant short-term goals.

Some discomfort is expected during both the home exercises and whole-body physical activity, however the pain should subside to usual levels by the next day with no increase in swelling following the exercise session. Participants will be taught how to determine whether pain levels during and for a short time after the exercises are acceptable. If a specific exercise is aggravating the participant’s pain, then the physiotherapist will reduce the resistance, dosage and/or level of challenge within the exercise until the pain flare settles.

#### Physiotherapy plus telephone coaching

The physiotherapy intervention will be the same as for the physiotherapy only group and delivered by the same physiotherapists. The participants in this group will also receive a telephone coaching intervention aimed at improving their adherence to their home exercise program and increasing their levels of general physical activity through behaviour change support. They will receive additional written information that explains the behaviour change support process. Telephone coaching sessions will be delivered 6–12 times during the 6-month intervention period. Calls will occur in weeks 2, 4, 8, 13, 21 and 25. Up to six additional calls can be made at any time during the 6 months according to participant confidence in taking action to change physical activity behaviours, adherence level, and preferences, as determined by the telephone coach, physiotherapist and participant. The initial call length is expected to range between 30–45 minutes with the remaining call durations lasting between 15 and 30 minutes.

The behaviour change model used for the telephone coaching intervention is the HCA Model of Health Change (http://www.healthchangeaustralia.com/the-hca-model.htm). Health Change Australia has been providing professional development training and consultancy in using the HCA Model of Health Change in Australia (since 2006) and Canada (since 2010). They have trained over 5,000 health professionals who work in a variety of public and corporate health settings. The HCA model has been used by both private and state based organisations to provide telephonic coaching services.

**Figure 2:**
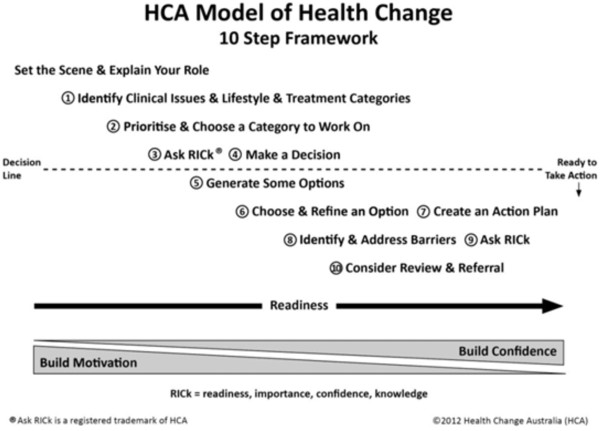
The HCA Model 10 Step Framework (used with permission from Health Change Australia).

The purpose of the HCA approach is to increase the likelihood that patients will act in accordance with lifestyle and treatment recommendations appropriate to their health condition/s, in this case OA. The model is comprised of 3 main components: a set of practice principles to guide communication and knowledge transfer, a set of essential techniques used to identify and address barriers to change and a 10 step decision framework that acts as a health behaviour change clinical pathway to guide clinical decisions (Figure 2). Effective use of the HCA Model also requires clinicians to have a foundation of 6 knowledge and skill sets. These are: health condition and health promotion knowledge, health behaviour change theory, health behaviour change interviewing skills, behaviour change facilitation skills, cognitive change facilitation skills and emotion-management facilitation skills. The first three knowledge and skill sets enable clinicians to effectively assist knowledge transfer in a way that makes it more likely that the patient will accept and use the information. The final three knowledge and skill sets provide the background required to flexibly apply the underpinning theoretical concepts and evidence-based techniques to facilitate behaviour change.

The nine practice principles represent critical aspects of the patient-centred approach. They act as prompts and tips to help clinicians build effective relationships and develop rapport with patients. The essential techniques are used to identify and address barriers to change. The first three are used to facilitate effective communication and so are used with every patient, however the remaining four are only used when particular barriers are present. The 10 step decision framework (Figure 2) represents ten prompts for clinicians to identify and address common ‘barriers to change’ that impact on patient motivation, commitment and confidence in taking action on treatment advice. Each step is associated with optional brief techniques that can be used to do this. The decision framework is used to help clinicians to systematically consider and work with a patient’s readiness, importance, confidence and knowledge (RICk) in relation to following treatment recommendations and lifestyle advice. Depending upon a client’s RICk profile, different health behaviour change processes and techniques are recommended for the clinician to apply to address behavioural, emotional, situational and thinking barriers to taking the prescribed actions.

The HCA approach enables the nurse telephone coaches to:

1. Provide participants with education and recommendations relevant to the program goals, -in this case specific exercises and increased general activity for OA management- in a way that reduces resistance and increases acceptance of this information.

2. Assist participants to make the decision that it is in their own interests to adopt the recommendations thus increasing client motivation and engagement in pursuing the behavioural goals.

3. Develop participants’ problem solving skills so that they are more systematic and successful in making decisions and initiating and sustaining physical activity behaviour changes.

The telephone coaches will check that participants understand that the best outcomes for OA are achieved by performing specific strengthening exercises, reaching recommended general activity levels, managing pain, and maintaining a healthy body weight. Specific emphasis will be placed on the first two of these behavioural treatment categories. If low motivation in either of these areas is detected, techniques will be used to help the participant to identify a personal motivator for taking the prescribed action. If the participant is ready to engage in the treatment recommendations and can see a personal benefit in adhering to the physiotherapist’s advice, then the telephone coach will proceed into goal setting and action planning to address any barriers identified. The nurse telephone coach will take into account the pain component of OA and its influence on physical activity behaviours by helping participants to incorporate principles of pain coping skills and learn about activity rest cycling (activity pacing). Managing lapses and relapses will also be discussed and contingency plans put in place. The individually tailored nature of the telephone coaching sessions will reinforce the initial goals set by the participant with their physiotherapist and allow these to be adjusted according to priorities and progress.

Transfer of information between the telephone coaches and physiotherapists is an important component in the successful delivery of the physiotherapy plus telephone coaching program. Following each physiotherapy and telephone coaching session, the physiotherapist or telephone coach will complete an on-line ‘Communication form’ outlining the topics discussed and problems experienced by the participant (if any), plus other relevant information such as functional goals identified, adherence to the program and other general physical activity plans. The telephone coach and physiotherapist will each be required to read the other’s entries prior to their session with the participant. This two-way interaction is designed to facilitate integration and consistency in this model of health service delivery.

### Treatment integrity

Study physiotherapists and telephone coaches will be provided with a detailed study procedures manual and will attend separate one-day training sessions on the specific study procedures.

The additional training for the coaches to deliver the telephone coaching intervention will involve attendance at the two-day HCA Core Training Part 1 workshop and the one-day HCA Core Training Part 2 workshop. The nurses will be mentored and required to practice their skills during the 3 months between HCA Core Training Part 1 and Part 2 and the study commencement. They will be given email and Skype access, and one face-to-face meeting with a HCA trainer (CB) during this training period. Each telephone coach will be provided with three practice patients not involved with the study in order to practice and develop their behaviour change support skills.

After trial commencement, online or telephone meetings will be held to discuss any issues experienced and solutions will be instigated. Physiotherapists and telephone coaches will keep standardised treatment notes and all telephone support calls will be recorded. A randomly selected 10% of telephone support calls will be audio-recorded and audited for adherence to the protocol and for quality of delivery by the study coordinator and HCA trainer (CB) respectively. All the calls made to the first two study participants allocated to each of the three telephone coaches will be reviewed and feedback on quality of health coaching will be provided to the coaches as further training and quality assurance. Participants will be questioned at the end of their treatment about their physiotherapy and telephone coaching (if applicable) treatment experience.

### Descriptive data

Height, weight and waist circumference will be recorded by the physiotherapist at the clinical screening visit and again at the final physiotherapy treatment session in Week 20. The baseline questionnaire booklet includes questions about age, gender, disease duration, medication use, prior treatments for knee pain and social circumstances.

### Outcome measures

#### Self-reported pain and physical function

The primary pain outcome is average knee pain during the past week. This, together with pain on walking during the past week, will be assessed using 11-point numeric rating scales (0 = no pain, 10 = worst pain possible). Such measurement has demonstrated reliability in OA [46]. Pain will also be assessed, along with physical function, using the disease-specific reliable and valid Western Ontario McMaster Universities (WOMAC) Osteoarthritis Index Likert version 3.1[47]. The pain subscale has five questions with five response options (0 indicating no pain, 4 indicating extreme pain) giving a total score out of 20, while the physical function subscale comprises 17 questions with five response options (0 indicating no physical dysfunction, 4 indicating extreme physical dysfunction) giving a total score out of 68. The latter will be used as the primary outcome measure of self-reported physical function.

At the follow-up assessments, participants will rate their perceived a) overall change, as well as change in b) pain and in c) physical function with the physical activity program (compared to baseline) on a seven-point ordinal scale (1-much worse to 7-much better). Scales of this kind are frequently used as an external criterion for comparison with changes in scores of other outcomes [48]. Measuring participant-perceived change using a rating of change scale has been shown to be a clinically relevant and stable method of identifying improvements that are truly meaningful from the individual perspective [49].

#### Physical activity

Physical activity will be measured using both self-report and objective techniques. The Physical Activity Scale for the Elderly (PASE) is a self-report questionnaire that has been shown to be reliable, valid and sensitive to change in people with knee OA [50,51]. It records both the level and type of recreational and occupational physical activity undertaken by participants over the previous week. The PASE was developed and validated in samples of older adults (age 55+ years) [52]. A second self-report tool, the Active Australia Survey [40], measures the time spent in physical activities and has acceptable reliability and validity in adult populations [41,53].

The activPAL ™ Professional will be used to objectively record physical activity levels. It consists of one small, lightweight sensor, and can monitor activity continuously for over seven days. The validity and reliability of the collapsed data have been previously established [54]. Inter-device reliability (intraclass correlation coefficient, ICC, 2,1) for time non-upright and standing has been reported as >0.99 and >0.99 respectively [55] and for walking at all speeds at >0.99 for both step number and cadence [56]. The mean difference between activPAL™ and observation ranged between 0.2% for total time non-upright and 3.7% for time standing [55]. It was developed for the purpose of categorising activity into either non-upright postures (incorporating sitting and lying) or upright postures (consisting of standing, transferring and walking). There is evidence of health benefits gained from being upright (standing and walking) despite the lower intensities of these activities [57] and given the aim of the intervention in this study was to achieve any increase in physical activity, including incidental walking, regardless of intensity, this device was the most appropriate of those available.

The activPAL™ will be worn continuously, except for when bathing or swimming, for at least seven consecutive days. The device samples the output from a single axis, gravity activated accelerometer at a frequency of 10Hz. It will be affixed to the skin overlying the mid anterior thigh with a re-usable gel PAL Stickie™, and further secured with a strip of Mefix® (Mölnlycke Health Care AB, Sweden) medical grade adhesive bandage. Custom-written software, using proprietary algorithms (Intelligent Activity Classification™, IAC™), categorises postures into standing, walking and non-upright positions (lying or sitting). The output provides the average total amount of time per day spent standing, the average total amount of time per day spent walking, and the average number of transitions from non-upright to standing positions per day.

#### Health-related quality of life

Health-related quality of life will be measured using the Assessment of Quality of Life instrument version two (AQoL2). The AQoL2 has 20 questions that cover six dimensions of health-related quality of life including independent living, social relationships, physical senses, coping, pain and psychological wellbeing. The AQoL2 has strong psychometric properties and is more responsive than other quality of life scales [58,59]. Scores range from −0.04 (worst possible health-related quality of life) to 1.00 (full health-related quality of life). A clinically important difference in health-related quality of life can be defined as a change of 0.04 AQoL units [60].

#### Other measures

Several other reliable and valid questionnaires that have been used in other OA and exercise studies will be administered to provide information about possible mediators of effects and effectiveness of implementation.

The *Arthritis Self Efficacy Scale* assesses confidence for managing pain (5 questions), physical function (9 questions) and other arthritis symptoms (6 questions) [61]. Responses to each question range from 1 (very uncertain) to 10 (very certain) with total scores ranging from 20 (lowest level of perceived self-efficacy) to 200 (highest level of perceived self-efficacy). Previous studies support the reliability and validity of this scale [61].

The *Self-Efficacy for Physical Activity Scale* evaluates confidence in one’s ability to participate regularly in physical activities with five questions regarding different feelings and situations [62].

The *Benefits of Physical Activity Scale* uses 14 questions to determine whether participants are aware of the benefits of physical activity and the *Barriers to Physical Activity Scale* uses 23 questions to identify which specific conditions make participation in physical activities difficult [63]. We will also use a short custom-designed questionnaire, similar to the Barriers to Physical Activity scale, that ask about barriers and enablers to performing the home exercises which are a key component of the intervention program.

The *Self-Regulation Scale* assesses the use of self-monitoring and goal setting strategies related to physical activity behaviour [64,65]. Twelve questions ranging from never (score = 1) to very often (score = 5) produce a range in total score from 12–60.

The *Arthritis Impact Measurement Scale Version 2 (AIMS2)* is a disease-specific self-reported instrument. Three of the 12 subscales, mood (five questions), tension (six questions) and thoughts of overall arthritis impact (1 question) will be used to assess psychological function in our OA participants. It has high-internal consistency, test-retest reliability and validity and it is moderately sensitive to change [66].

The *Depression, Anxiety and Stress Scale (DASS)* measures three negative emotional states of depression, anxiety and stress over the previous week [67]. The 21-item short-form consists of seven questions for each emotion. Responses range from “0” (did not apply to me) to “3” (applied to me very much, or most of the time) and scores from each subscale are summed and multiplied by two to give a total score in the range of 0–42. Higher scores indicate greater levels of distress. It has high internal consistency and construct validity [67,68].

The *Brief Fear of Movement Scale* incorporates 6-items using a four point scale from “strongly agree” to “strongly disagree” to assess fear of injury/reinjury due to movement [69]. It has sound psychometric properties and consistent performance across diverse groups of individuals with hip and knee OA.

The *Patient Health Questionnaire-9 (PHQ-9)* is a 9-item depression scale that scores symptom severity on each of the 9 Diagnostic and Statistical Manual of Mental Disorders, Fourth Edition (*DSMIV*) criteria. Responses to each question range from 0 (not at all) to 3 (nearly every day) with the sum of scores therefore ranging from 0–27. Scores of 15 or greater represent moderately severe to severe depression. It is commonly used in clinical settings and is a reliable and valid measure of depression severity [70].

We will use the *Coping Strategies Questionnaire (CSQ)* to assess the use of pain coping skills [71]. This 50-item scale measures how often a patient engages in seven different pain coping strategies (six cognitive responses and two behavioural responses), plus two questions on their perceived control over their pain and their ability to decrease their pain based on their use of coping strategies. This instrument has demonstrated sensitivity to change from treatment in chronic pain samples as well as good internal consistency and construct validity [72].

Pain catastrophising will be measured using the 13-item *Pain Catastrophising Scale* which measures tendencies to ruminate about pain, magnify pain, and feel helpless about pain on scales from 0–4. The highest possible total score of 52 indicates the greatest level of catastrophising. It has high internal consistency and is associated with heightened pain, psychological distress, and physical disability [73].

#### Adherence

The number of physiotherapy visits and telephone coaching calls, as well as the timing and duration of the calls, will be recorded. At 3, 6, 9, 12, 15 and 18 months, participants in both groups will be asked by postal questionnaire how many times they did their home exercises in the previous 2 weeks (out of a maximum of 6 sessions). They will also be asked to rate their adherence to their home exercise program over the previous 3 months (from ‘not at all’ to ‘completely as instructed’) using an 11-point numeric rating scale and to rate their change in physical activity level since the start of the study using a 7-point Likert scale (from ‘much less’ to ‘much more’. Physiotherapists and telephone coaches will also rate the participant’s adherence to the 6-month program using an 11-point numeric rating scale.

#### Adverse events, harms and use of health services/co-interventions

Information on adverse events, harms and direct health care costs and direct non-health care resources will be collected for each 3 month period during the study using log sheets given prospectively and collected at 3, 6, 9, 12, 15 and 18 months. Missing log sheets will prompt a phone call to collect the data retrospectively. Harms are adverse events that can be attributed to the intervention (home exercise program or additional physical activity). The adverse event log sheet will ask duration (days) of the problem and whether any treatment was sought for the problem regardless of cause.

Direct health care costs will include costs of physiotherapy attendance, additional health provider visits (doctors, specialists, other health care professionals), investigative procedures, purchase of prescription and over the counter medication, and hospitalization. Direct non-health care resources will include number of lost days from work.

### Economic Evaluation

The economic evaluation will assess the incremental cost of the physiotherapy plus telephone coaching intervention compared with physiotherapy only. The primary economic evaluation will report cost effectiveness at 18 months with secondary economic evaluations at 6 and 12 months. The incremental cost will be compared to the incremental benefits of treatment in terms of a clinically significant improvement in pain, a clinically significant improvement in function, and the difference in quality adjusted life years (QALYs). The incremental QALYs will be measured by the between group difference in the mean AQoL2 score over 6, 12 and 18 months. A social perspective on costs will be taken and will include resource use incurred both by health services and by the patient irrespective of payment source. Prospective self-reported direct health care use will be collected every 3 months using log sheets. Health care costs will be calculated from the utilisation data and published average unit costs for each item. The direct cost of the intervention will be based on physiotherapy charges per session and telephone coaching costs per person. The inclusion of time/productivity gains is controversial and the cost effectiveness ratios will be calculated with and without these “indirect costs”. Confidence intervals for incremental cost effectiveness will be calculated directly using non parametric bootstrapping. In addition we will calculate a cost effectiveness acceptability curve based for a range of hypothetical money values of outcomes [74]. This will be done using individual cost and outcome data over the 6, 12 and 18 months or, if adjustments for imbalance at baseline are necessary, using regression analysis [75]. As part of the economic evaluation we will survey participants at the 6, 12 and 18 month questionnaire about their satisfaction with the intervention including its value for money.

### Sample size

The primary endpoints will be change from baseline to 6 months in (i) knee pain (NRS) and (ii) physical function (WOMAC). The minimum clinically important difference to be detected in OA trials is a change in pain of 1.8 units on NRS [76] and a change in physical function on WOMAC of 6 units (out of 68) [77]. Randomisation is stratified by physiotherapist and hence no clustering effects of participants within physiotherapists need be accounted for. However, there may be clustering effects due to participants treated by the same telephone coach in the physiotherapy plus telephone coaching arm which we account for as follows: With three telephone coaches, one third of the participants in the physiotherapy plus telephone coaching arm are expected to be treated by each coach, and we assume in the sample size calculation an intra-cluster correlation of 0.05, which has been demonstrated to be conservative for patient-level outcomes across health care practices [78]. Based on combined data from our 12 month RCT in 200 people with knee OA [79] and our 12 week RCT in 76 people with knee OA who undertook a hip strengthening program [22], we assume a between-participant standard deviation of 2.2 for pain and 11.6 for WOMAC physical function, and a baseline to 6-month correlation in scores of 0.29 for pain and 0.51 for physical function. These assumptions, together with an analysis of covariance adjusted for baseline scores and for clustering, produce a sample size of 67 patients per intervention arm to achieve 80% power to detect the above differences. Allowing for a 20% attrition rate we will recruit 84 patients per arm, or 168 patients in total.

### Data and statistical analysis

A biostatistician (AF) will oversee the blinded analyses of the data. Main comparative analyses between groups will be performed using an intention-to-treat analysis. To account for missing data, multiple imputation of missing follow-up measures, assuming missing data are missing at random and follow a multivariate normal distribution [80] will be performed as a sensitivity analysis. For continuous outcome measures, differences in mean change (baseline minus follow-up) will be compared between groups using linear regression random effects modelling adjusted for baseline values of the outcome and clustering effects of telephone coaches. Model diagnostic checks will utilise residual plots. Similar regression models for binary and ordinal outcome measures will use random effects logistic and proportional odds models, respectively. Estimates of intervention effects under hypothetical full adherence will be performed using recently developed methods based on causal modelling ideas [81] which we have successfully applied to data from our recently completed knee OA trial [79].

### Timeline

Ethics approval was obtained in May 2012 from the Human Research Ethics Committee of the University of Melbourne. Recruitment and training of the health coaches occurred during March – May 2012, and training of the physiotherapists was carried out in May 2012. Recruitment of participants commenced in June 2012. All participants are expected to have completed the study by end 2015.

## Discussion

The need to develop efficacious treatment approaches for knee OA that achieve long-term sustainability of improved outcomes is an important research and clinical objective. As there is no cure for OA, lifestyle behavioural change, particularly in the area of physical activity, is pivotal in knee OA management. This pragmatic RCT will provide internationally relevant, high quality Level 2 evidence of the longer-term clinical- and cost-effectiveness of a physical activity intervention involving a limited number of physiotherapy visits with and without telephone coaching. Furthermore, our inclusion of regional participants enhances the generalisability of results.

Our study will provide novel information about a clinical practice model for behaviour change support delivered by telephone that targets specific physical activity behaviours rather than a number of generic self management messages as the limited previous studies in knee OA have done. This does not diminish the importance of other aspects of self-management but rather allows interpretation of the specific effects of a physical activity intervention. The identification of feasible health care models to facilitate physical activity behaviours and improve outcomes in people with knee OA has important implications for clinical practice. Economic evaluation of treatment is also crucial in today’s health care landscape. The inclusion of this aspect in our RCT offers an additional dimension that will assist health policy makers in their decision-making regarding funding.

Telephone-delivered interventions have the potential to be adopted by the growing number of government and non-government agencies and health funds that operate telephone information and support centres. If the results of our proposed study support the clinical- and cost-effectiveness of a physiotherapy and telephone coaching intervention for knee OA, the program could be easily implemented into clinical practice using a standardised model of care. We deliberately designed the study to test an easily implementable service delivery model so as to maximise translatability. This was done by using a limited number of physiotherapy contacts consistent with the number available under the existing Medicare scheme, by using a home-based exercise program suitable to a broader public health approach, and by using a transferable telephonic coaching intervention that is inexpensive, widely accessible by and acceptable to this older patient population. Such a physical activity intervention could benefit the large number of people with knee OA and would be particularly useful for those in regional areas who may not be able to access other services.

Our study is the first RCT to investigate the effect of a telephone coaching intervention to specifically support a physiotherapist-delivered physical activity intervention on pain and function in people with knee OA. Strengths of the study design are the pragmatic nature of treatment delivery by practicing physiotherapists in community physiotherapy clinics, and by health practitioners trained in the HCA model of Health Change, as well as the reproducibility of both the physiotherapy and telephone coaching programs. These features will improve the ability to translate the findings into a range of interdisciplinary health care settings and enable future researchers to replicate the behaviour change intervention. Importantly the physical activity program is individualised with regard to the content and intensity level of the home exercises and the plan for increasing daily levels of physical activity. The study is designed so as to conform to CONSORT requirements for non-pharmacological interventions. It is adequately powered for our primary outcome measures and our recruitment strategy will result in a well-characterised sample from both metropolitan and regional areas. In addition, our study includes longer-term follow-up and primary outcomes recommended by international osteoarthritis researchers and which are of relevance to the recipients of the intervention.

## Competing interests

The authors declare that they have no competing interests.

## Authors' contributions

KLB and RSH conceived the project; KLB procured the project funding and is leading the co-ordination of the trial. KLB, RSH, TE, JG, CB, GSK, SJB, DJH, and CAB assisted with protocol design. KLB, RSH and TE designed the physiotherapy program and, along with CB, trained the physiotherapists. JG and CB trained the clinicians in the HCA Model of Health Change and HCA provided their manualised protocols for the behaviour change support component. KLB, CB and TE provided study-specific training for the telephone coaches, CB provided mentoring and feedback during the nurse telephone coach’s training and practice phase and HCA provided quality auditing tools matched to the manualised telephone coaching protocols. TE wrote the study procedures manual, the Information and Education Booklet, and the first draft of this manuscript. KLB, RSH, CB and TE completed the writing of the manuscript. AF performed the sample size calculations and designed the randomisation schedule and statistical analyses. AH designed the methods for the economic analysis. All authors participated in the trial design, provided feedback on drafts of this paper and read and approved the final manuscript.

## Pre-publication history

The pre-publication history for this paper can be accessed here:

http://www.biomedcentral.com/1471-2474/13/246/prepub
